# The pathogenic mechanism of monosodium urate crystal-induced kidney injury in a rat model

**DOI:** 10.3389/fendo.2024.1416996

**Published:** 2024-07-01

**Authors:** Delun Li, Yimeng Li, Xuesheng Chen, Jianting Ouyang, Danyao Lin, Qiaoru Wu, Xinwen Fu, Haohao Quan, Xiaowan Wang, Shouhai Wu, Siyu Yuan, Anqi Liu, Jiaxiong Zhao, Xiaowu Liu, Gangxing Zhu, Chuang Li, Wei Mao

**Affiliations:** ^1^ State Key Laboratory of Dampness Syndrome of Chinese Medicine, The Second Affiliated Hospital of Guangzhou University of Chinese Medicine, The Second Clinical College of Guangzhou University of Chinese Medicine, Guangzhou, China; ^2^ Department of Nephrology, The Second Affiliated Hospital of Guangzhou University of ChineseMedicine (Guangdong Provincial Hospital of Chinese Medicine), Guangzhou, China; ^3^ State Key Laboratory of Dampness Syndrome of Chinese Medicine, The Second Affiliated Hospital of Guangzhou University of Chinese Medicine, Guangzhou, China; ^4^ Nephrology Institute of Guangdong Provincial Academy of Chinese Medical Sciences (NIGH-CM), Guangzhou, China; ^5^ Ministry of Education Key Laboratory of Pharmacology of Traditional Chinese Medical Formulae, Tianjin University of Traditional Chinese Medicine, Tianjin, China; ^6^ School of Chinese Materia Medica, Tianjin University of Traditional Chinese Medicine, Tianjin, China; ^7^ State Key Laboratory of Component-based Chinese Medicine, Tianjin University of Traditional Chinese Medicine, Tianjin, China; ^8^ Cadre Department, Guizhou Provincial People’s Hospital, Guizhou, China

**Keywords:** MSU crystal, uric acid, gouty nephropathy, hyperuricemic nephropathy, model

## Abstract

**Objective:**

(MSU) crystals usually in the kidney tubules especially collecting ducts in the medulla. Previous animal models have not fully reproduced the impact of MSU on kidneys under non-hyperuricemic conditions.

**Methods:**

In the group treated with MSU, the upper pole of the rat kidney was injected intrarenally with 50 mg/kg of MSU, while the lower pole was injected with an equivalent volume of PBS solution. The body weight and kidney mass of the rats were observed and counted. H&E staining was used to observe the pathological damage of the kidney and to count the number of inflammatory cells. Masoon staining was used to observe the interstitial fibrosis in the kidneys of the rat model. Flow cytometric analysis was used for counting inflammatory cells in rats. ElISA was used to measure the concentration of serum and urine uric acid, creatinine and urea nitrogen in rats.

**Results:**

At the MSU injection site, a significantly higher infiltration of inflammatory cells and a substantial increase in the area of interstitial fibrosis compared to the control group and the site of PBS injection were observed. The serum creatinine level was significantly increased in the MSU group. However, there were no significant differences in the rats’ general conditions or blood inflammatory cell counts when compared to the control group.

**Conclusion:**

The injection of urate crystals into the kidney compromised renal function, caused local pathological damage, and increased inflammatory cell infiltration and interstitial fibrosis. Intrarenal injection of MSU crystals may result in urate nephropathy. The method of intrarenal injection did not induce surgical infection or systemic inflammatory response.

## Introduction

Gouty nephropathy (GN) is a renal condition caused by precipitation of monosodium urate (MSU) crystals in the kidney tubules especially collecting ducts in the medulla (1). Hyperuricemia is prevalent in China, with a notable prevalence of 13.3% (2). The persistent presence of supersaturated level of uric acid (UA) in biological fluids causes the formation of MSU crystals in the kidneys. A subnormal pH also promotes precipitation of MSU crystals as the first pKa value of uric acid is about 5.6 (3, 4). Mechanistically, following phagocytosis, intracellular MSU crystals via activation of the NLRP3 inflammasome complex in monocytes/macrophages stimulates the secretion of IL-1β, IL-18 etc. into the extracellular space that cause recruitment of leucocytes from the bloodstream into the inflamed site of the tissue (3, 5, 6). MSU crystals were also shown to directly activate neutrophils (7).

Generally, it was believed that asymptomatic hyperuricemia is less likely to cause kidney injury. However, recent research indicates that asymptomatic hyperuricemia can actually induce inflammation and consequently lead to kidney injury (7, 8). This new finding suggests that animal models previously used for understanding the cause of GN were likely simulating hyperuricemic nephropathy (HN) instead of GN. The majority of these prior models employed various methodologies to elevate blood uric acid levels, such as raising blood uric acid, by inhibiting uric acid excretion (9), and inhibiting hepatic uricase that converts uric acid into more soluble allantoin (10). Rat models elicited by these methods were primarily used to simulate renal injuries caused by hyperuricemia. Although lengthening the modeling period may result in MSU deposition in the kidneys, which could cause injury, it is unclear whether the causal factors are hyperuricemia, MSU, or a combination of both. Therefore, in these models, it is impossible to know the impact of only deposited MSU on kidneys under the condition of normal blood urate level.

In this study, we have introduced a new approach that involves the intrarenal injection of MSU to induce GN. The aim is to elucidate the pathological impact of MSU on the kidney. This approach could potentially serve as an experimental groundwork for future research on the pathogenesis and prevention strategies of GN.

## Materials and methods

### Animals

The use of animals in this study was approved by the Animal Policy and Welfare Committee of Guangdong Academy of Chinese Medicine (Approval Document No. 2022051) and all animal experiments were conducted in accordance with the guidelines of the National Institutes of Health (NIH). The experiments were also performed in accordance with NIH guidelines. Male SD (Sprague Dawley rat) rats were purchased from Charles River and placed in an SPF(Specific-pathogen-free)-grade environment (SPF) with a relative humidity of 40% to 70%, an ambient temperature of 20–25°C, and a photoperiod of 12/12 h.

### Experimental grouping

**Figure 1:**
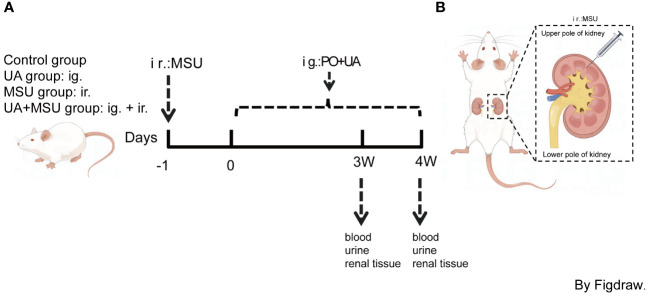
Flowchart of strategic study design in animal model (rat). **(A)** Control, uric acid (UA), MSU and UA+MSU groups: For the UA group, the 8-week-old male rats were gavaged (intragastric; ig) with 750 mg/kg potassium oxonate (PO) and 300 mg/kg uric acid once daily. For the MSU group, the suspension of MSU crystals (10 mg/100 μl/kidney) was injected (intrarenal; ir) with an injection needle into the renal cortico-medullary junction at the upper pole of the kidneys of the 8-week-old male rats. An equal volume (100 μl) of PBS (pH 7.4) was injected into the lower pole of the kidney for site-specific control of the MSU-injected kidney. For the UA+MSU group, the suspension of MSU crystals (10 mg/100 μl/kidney) was injected (ir) into the renal cortico-medullary junction at the upper pole of the kidneys of the 8-week-old male rats that were gavaged (ig) with PO + UA as described above on the first day after the MSU injection. An equal volume (100 μl) of PBS (pH 7.4) was injected into the lower pole of the kidney for site-specific control for UA+MSU effect. Urine samples, blood samples and kidney tissues were collected three or four weeks (3W/4W) after injection. **(B)** MSU suspension was injected into the upper pole of the kidney as shown in the figure. MSU, monosodium urate crystal.

After one week of acclimatization, the rats were randomly divided into a control group, a GN group (MSU group), a HN group(UA group), and a Joint GN and HN modelling group (UA+MSU group). Each group was further divided into two observation time points, at the third and fourth weeks, respectively. Each group consisted of eight rats. GN was induced in the rats by injecting urate crystals into their kidneys. HN was induced by intragastric administration 750 mg/kg potassium oxonate (PO; a competitive inhibitor of uricase) (Cat # 2207–75-2, Sigma-Aldrich) and 300 mg/kg uric acid (Cat # A8626 Sigma-Aldrich). For combined GN and HN model group MSU crystals were injected into kidneys the day before gavage of a mixture of PO and uric acid. The administrationof PO and uric acic was continued for 3 or 4 weeks (**Figure 1A**). To anesthetize the rats, chloral hydrate (350 mg/kg, i.p) was administered intraperitoneally. The hair on the rats’ backs below the sternum was removed and disinfected with iodophor. The rats were considered suitably anesthetized when they exhibited general weakness or responses. The rats were positioned supine on a sterile operating table. The epidermis and muscle layers were incised using surgical scissors, and the abdomen was gently compressed to extrude the kidney from the abdominal cavity through the dorsal surgical opening. The MSU crystals were administered with an injection needle at the renal cortico-medullary junction at the upper pole (suprarenal pole) of the rat kidney, approximately 1.3–2/3 of the width of the rat kidney (i.e. 5–10 mm). The injection volume was 100 µl (0.1 mg/µl) per kidney, and the needle was removed after the injection. An equal volume of Phosphate buffered saline (PBS) (AR0032, Bosterbio Biotechnologies) was injected into the lower pole (infrarenal pole) of the kidney. The kidneys were replaced in the abdominal cavity and replenished with 1 ml of saline. Finally, the muscle and epidermal layers of the rats were closed using sutures. After suturing, the wounds were cleaned with saline cotton pads to remove any blood. The rats were then placed on a heating plate and returned to their cage once they had awoken. The rat model of GN induced by MSU was successfully obtained.

### Preparation of MSU crystals for injection

Uric acid was dissolved in pure water with the help of 3M NaOH added dropwise until complete dissolution. The pH was adjusted to 8.9 using NaOH and glacial acetic acid. The uric acid was then left to crystallize for 2 days at room temperature. The crystals were collected on filter paper, washed 3 times with 70% ethanol, allowing to dry at 37–45°C. MSU crystals were collected in centrifuge tubes and autoclaved at 120°C and 15 psi for about 30 min. A sterile PBS solution was used to prepare the MSU crystal suspension.

### H&E staining

The kidneys were fixed in a 4% paraformaldehyde solution and trimmed to a suitable shape using a scalpel. Subsequently, the tissue was dehydrated using alcohol of increasing concentration. The dehydrated tissue was then dipped in wax to get paraffinembedded tissue blocks. The chilled paraffin-embedded tissue blocks are sectioned into 4 µm thin sections. The paraffin sections of rat kidneys on glass slides were dewaxed in Xylene, dehydrated by washing in absolute ethanol and then washed in water for 3–5 minutes. After deparaffinization, dehydration and subsequent hydration, slides were immersed in water and stained with the H&E Staining Kit (HEC005M, Beyotime). The slides containing hydrated tissue sections were completely submerge in hematoxylin staining solution for 15–20 min until the nuclei were clearly stained. The sections were then washed in PBS buffer with sufficient swirling and stained with Eosin. The extent of the staining was observed under the microscope. Finally, the tissue sections were allowed to dry and sealed with a neutral resin before observing and photographing the morphological changes in the rat kidney under a light microscope. We randomly selected 10 fields of view for the sections of whole rat kidneys in the control group, and 5 fields of view each for the sections of upper and lower poles of the kidney of rats in the GN group.

### Masson’s Trichrome staining

The Masson (Trichrome) staining (used primarily to demonstrate connective tissue elements, collagen and muscle fibers) of the kidney sections, involves fixing, dehydrating, embedding, and sectioning in order before staining using the Masson staining kit (DC0033, Leagene Biotechnology). After staining, collagen becomes colored blue, muscle fibers, cytoplasm and keratin in red and the nuclei blue/black. The staining of the tissue sections was performed in the order of Bouin’s solution (an excellent fixative for preserving soft and delicate structures), azurite blue staining solution, hematoxylin staining solution (to stain nuclei), color separation solution, and Rejuvenate red staining solution. Phosphomolybdic acid solution was used for staining connective tissue fibers to Aniline Blue. Finally, the sections were dried and sealed with resin. The level of fibrosis in rat kidney tissue was observed and photographed under a microscope. In the control group, 10 random fields of view were selected from the entire kidney tissue, while in the GN group, 5 random fields of view were selected from each of the suprarenal and infrarenal poles. The positive area was counted by analyzing the collected fields of view using Image J software.

### Immunohistochemistry

Kidney sections were formalin fixed, dehydrated, sectioned into 4µm thickness and then embedded on glass slides. For heat induced antigen retrieval, tissue sections fixed on slides were dipped in 10mM sodium citrate solution (pH 6.0) (AR0024,Bosterbio Biotechnologies) and heated in a pressure cooker for 4 minutes. Endogenous peroxidase enzymes in the tissue sections were inhibited by dipping the tissue-embedded slides in 3% H202 solution for 15 minutes. The slides were then blocked with 5% BSA (bovine serum albumin) in PBS (phosphate-buffered saline)solution (180728, Genxion Biotechnologies) for 1 h at room temperature to minimize non-specific binding of primary and secondary antibodies before incubating with the appropriate primary antibody (diluted in 1% BSA in PBS) for twelve hours at 4°C. The tissue sections embedded on slides were washed three times with PBS and stained appropriate secondary antibody (KIT-5020, MXB Biotechnologies) for one hour at 25°C. The tissue sections were then washed with PBS to remove non-specifically adhering the secondary antibodies and then stained using 3,3’-diaminobenzidine (DAB) (DAB 2031, MXB Biotechnologies) to develop dark-brown color by hydrogen peroxide in the presence of HRP-linked secondary antibody attached to the primary antibody which is bound to the antigen. After drying the sections, we used neutral resin to seal them before observing and photographing the morphological changes in the rat kidney under a light microscope. We randomly selected 10 fields of view for the sections of whole rat kidneys in the control group, and 5 fields of view each for the sections of upper and lower poles of the kidney of rats in the GN group.

To identify crystal deposits in the kidneys, paraffin sections of kidneys from mice on the day 28 were stained with hematoxylin and eosin (H&E) and UA crystal deposits were visualized under polarized light.

Multiplex fluorescence immunohistochemistry was conducted in accordance with the instructions provided in the staining kit (G1236–50T, ServiceBio), utilizing paraffin sections of rat kidneys and TSA-488 dye-conjugated NLRP3 antibody, TSA-555 dye-conjugated F4/80 antibody, and TSA-647 dye-conjugated CD11b antibody. The stained sections were then subjected to tissue panoramic scanning using a tissue panoramic scanner(VS200,PanoView).

Antibody: Anti-α-smooth muscle actin antibody (a-SMA)(Cat # ab124964, abcam); anti-Vimentin antibody (Cat # ab92547, abcam) for cytoskeleton detection; anti-NLRP3 antibody (Cat # T55651, Abmart), anti-integrin αM/CD11b antibody (Cat # sc-1186, Santa Cruz); anti-F4/80 antibody (Cat # ab300421, abcam) for macrophage detection.

### Uric acid dissolution test

A 50X solution (50 mM, pH 9.0) of ethylenediaminetetraacetic acid (EDTA; P0084, Beyotime) was diluted to 1X (1 mM, pH 9.0) using ultrapure water. One milligram of uric acid oxidoreductase (S10175, Yuanye Bio) was volumized to 1 ml of 1X EDTA solution to formulate a uric acid oxidase (UOX) solution at a concentration of 37.87 u/ml. The prepared MSU crystals were placed on slides and the UOX solution or EDTA solution (as a negative control) was added dropwise. Following the dropwise addition of the solutions, observations were conducted under a microscope at 200x magnification at 0, 5, 10, 20 and 30 minutes.

### Analysis of circulating inflammatory cell counts

The rats were anesthetized with chloral hydrate (350 mg/kg, i.p) and confirmed the depth of anesthesia suitable for surgery by monitoring their heart rate, respiratory rate, blood pressure, weakness and unresponsiveness. The abdominal cavity was then opened to expose the abdominal aorta and blood was drawn using a blood collection needle and collected in vial containing heparin (EDTAK2,SANLI CHINA) and sent to the the Laboratory Department of Guangdong Provincial Hospital of Traditional Chinese Medicine, University Town Branch, for testing. The blood was analyzed using a fully automated blood cell analyzer (Myriad, BC-6000Plus).

### Test of renal functioning

Glomerular filtration rate (GFR) is the best overall index of kidney function. The value of GFR is calculated from the concentration of creatinine and blood urea nitrogen (BUN) in serum and in urine, respectively. Therefore, urine was collected from each rat in a metabolic cage for 24 hours prior to observation. Blood samples were collected as described above, centrifuged, and the upper serum layer was separated. The levels of uric acid (Cat # C012–2-1, Nanjing Jiancheng Bioengineering Insitute), creatinine (Cat # C011–2-1,Nanjing Jiancheng Bioengineering Insitute), and blood urea nitrogen (BUN) (Cat # C013–2-1, Nanjing Jiancheng Bioengineering Insitute) in both serum and urine were measured following supplied protocols of the respective appropriate kits.

### ELISA

The concentration of IL-1β in the serum was measured using the ELISA kit for rat IL-1β (Cat # CSB-E08055r, Cusabio) following manufacturer’s supplied protocol. The absorbances (Optical Density, OD) of ELISA tests were read at 450 nm.

### Statistical analysis

Statistical analysis was conducted using GraphPad Prism 8.0 (GraphPad Software Inc., San Diego, CA, USA). The data were presented as mean ± SEM. The normality of the data (relative value of the SD with respect to mean) was confirmed, and group comparisons were assessed using either one-way ANOVA or Student’s t-test. A p-value of less than 0.05 was considered statistically significant. The symbol” * “ is employed to indicate statistical differences from the control group, the symbol “ # “ is employed to indicate statistical differences between any two groups, with the exception of the control group.

## Results

### Intrarenal administration of MSU crystal suspension shows no impact on the whole body weight and kidney mass of rats

**Figure 2:**
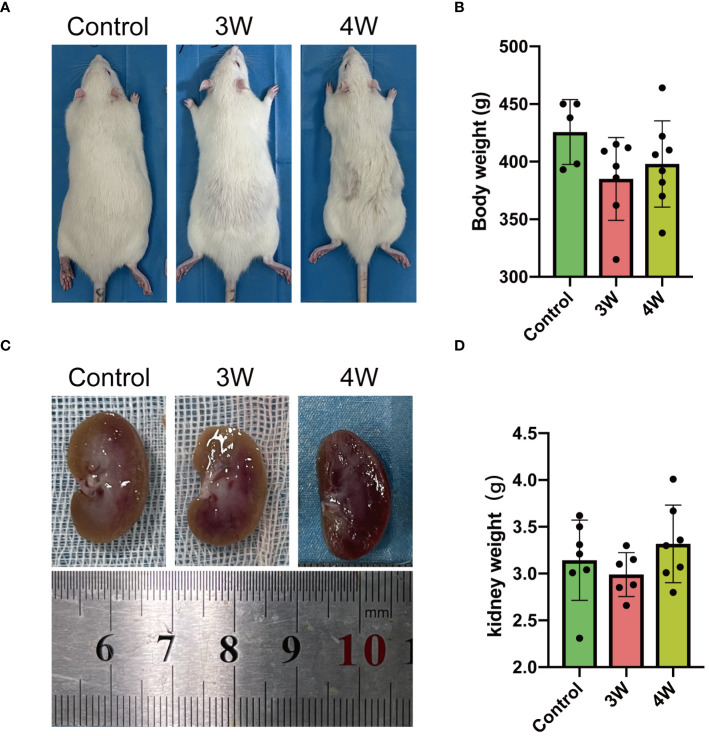
Intrarenal administration of MSU crystals appears to have no significant effect on physical appearance of the animals (rats), whole body weight and outward appearance and mass of kidney. **(A)** The pictures of the rats were taken at three or four weeks (3W/4W) after injection of suspension of MSU crystals. **(B)** The whole body weight (g) of rats were measured three or four weeks after injection of suspension of MSU crystals. **(C)** The pictures of the physical appearance of rat kidneys taken at three or four weeks after injection of suspension of MSU crystals. **(D)** The weight (g) of rat kidneys was measured at three or four weeks after injection of suspension of MSU crystals.

The outward appearance of the rat kidneys and the mass were compared across two different groups post-injection. The results show that intrarenal injection of MSU crystals did not significantly alter the physical appearance (**Figure 2A**), whole body weight (**Figure 2B**) and overall appearance (**Figure 2C**) or weight (**Figure 2D**) of the kidneys compared to controls during the third and fourth weeks after the injections. Thus, intrarenal injection of MSU crystals seems did not affect the overall health conditions of rats in the third and fourth weeks post-procedure.

### Intrarenal MSU injection caused localized renal tubular damage and an increase in inflammatory cell infiltration

**Figure 3:**
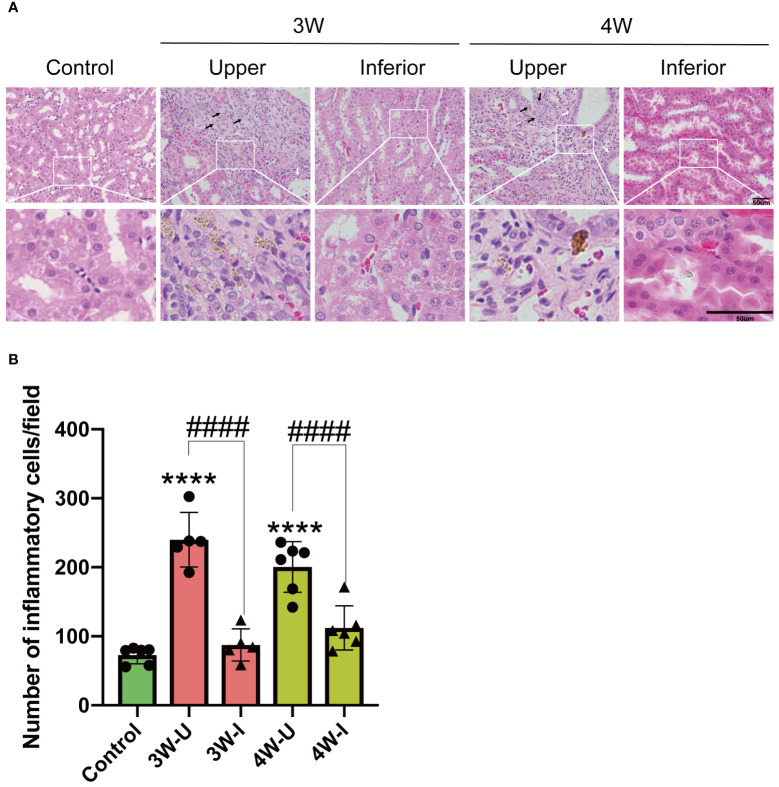
Intrarenal administration of MSU crystals exhibited significant damage to the peripheral renal tubules; characterized by tubular dilatation,vacuolar degeneration and substantial infiltration of inflammatory cells into the renal interstitium. **(A)** The Hematoxylin and Eosin (H&E) staining of a representative of the kidney sections (n = 6; mean ± SEM) of the MSU crystals injected upper pole and PBS injected lower pole. Scale bar = 50 μm. Arrow sign (→) on the picture shows damage to the peripheral renal tubules, the white arrow indicates tubular dilatation, while the black arrow denotes vacuolar degeneration. **(B)** Inflammatory cell count infiltrated into the interstitium of rat kidneys three or four weeks (3W/4W) after injection of suspension of MSU crystals in the upper pole (3W-U/4W-U) and PBS injection in the lower pole (3W-I/4W-I) of the rat kidney. (n = 5–6; mean ± SEM). **** P < 0.0001. #### P < 0.0001.

**Figure 4:**
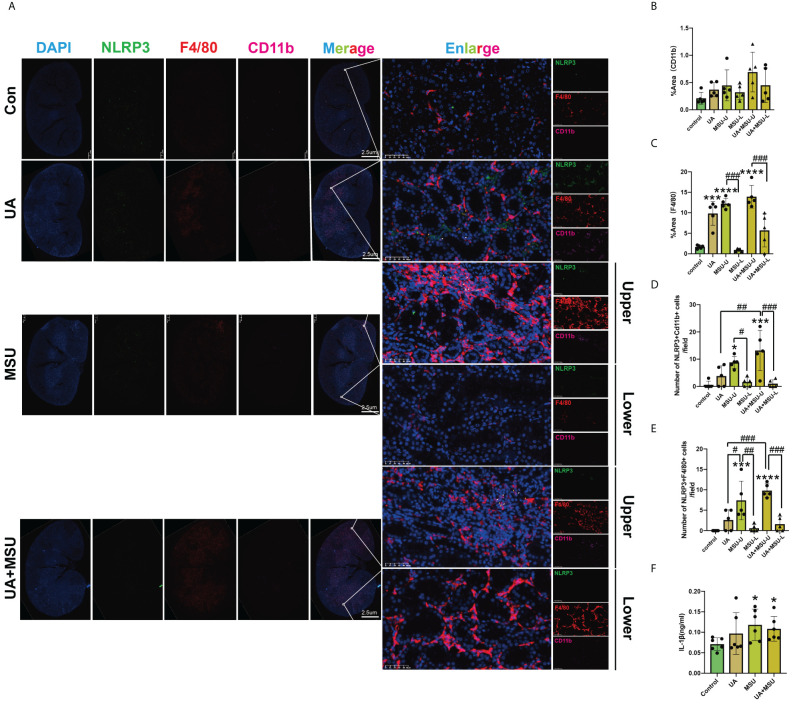
Intrarenal injection of MSU crystals into the kidneys of normal rats or PO-induced hyperuricemic rats induces the activation of NLRP3 inflammasome protein complexes in renal monocytes and macrophages and elevate serum IL-1β levels. **(A)** Representative images of kidney sections of control rat, PO-induced hyperuricemic rat (UA group), MSU crystals-injected rat (MSU group), and MSU crystals-injected PO-induced hyperuricemic rat (UA+MSU group) at 28 days of after MSU injection/gavage that were stained using TSA-488 dye-conjugated NLRP3 antibody, TSA-555 dye-conjugated F4/80 antibody, and TSA-647 dye-conjugated CD11b antibody for Multiplex immunohistochemical staining. Green fluorescence represents NLRP3, red fluorescence represents F4/80, and pink fluorescence represents CD11b. White arrows represent double-positive cells (macrophages and monocytes) for NLRP3+CD11b+; yellow arrows represent double-positive cells (macrophages) for NLRP3+F4/80+. Scale bars = 2.5 μm and 50 μm. **(B)** Quantification of the pink area immune-stained for CD11b (n = 5; mean ± SEM). **(C)** Quantification of the read area immune-stained for F4/80 (n = 5; mean ± SEM). **(D)** The relative number of double-positive cells for NLRP3+CD11b+ per view (n = 5; mean ± SEM). **(E)** The relative number of double-positive cells for NLRP3+F4/80+ per view (n = 5; mean ± SEM). **(F)** The concentrations of IL-β in the sera of all four groups of rats (n = 5; mean ± SEM). Rat kidneys were sectioned four weeks (4W) after MSU injection/gavage. Blood samples from all four groups of rats (for sera) were collected four weeks after MSU injection/gavage. *, # <0.05; **, ## P < 0.01; ***, ### P < 0.001; ****, P < 0.0001 The panoramic scanning of slides of kidney sections stained for multiplex fluorescence immunohistochemistry with TSA-488 dye conjugated NLRP3 antibody, TSA-555 dye conjugated F4/80 antibody (for labeling matured macrophages), and TSA-647 dye conjugated CD11b antibody (for labeling monocytes, activated lymphocytes, macrophages) was used for counting the number of infiltrated inflammatory cells.

To examine the impact of intrarenal MSU crystals on renal pathology, we conducted Hematoxylin and Eosin (H&E) staining of renal tissue sections. The results revealed the presence of uratoma at the upper pole of the kidneys injected with MSU crystals by the end of third and fourth weeks post-injection (**Figure 3A**). The MSU crystals exhibited significant damage to the peripheral renal tubules, characterized by tubular dilatation and vacuolar degeneration (White arrows and black arrows). In contrast, the lower renal pole injected with PBS revealed no significant pathological changes like the control group, with no discernible damage at weeks three and four (**Figure 3A**). MSU crystals were shown to provoke pathological damage at its location by inducing innate immunity (11). To know the mechanism of how MSU crystals induce renal tissue inflammation, we measured the number of infiltrated inflammatory cells (cellular components of innate immunity) into the interstitium of renal tissues (**Figure 3B**). The results of panoramic scanning of slides of kidney sections stained for multiplex fluorescence immunohistochemistry with TSA-488 dye conjugated NLRP3 antibody, TSA-555 dye conjugated F4/80 antibody (for labeling matured macrophages), and TSA-647 dye conjugated CD11b antibody (for labeling monocytes, activated lymphocytes, macrophages) revealed a significant increase in inflammatory cell infiltration at the upper renal pole injected with MSU crystals during weeks four, compared to the PBS-injected and control kidneys (**Figures 3B**, **4A–C**). We did not find any significant increase in infiltration of inflammatory cells in the PBS-injected lower renal pole compared to the control kidneys during these time periods.

### Intrarenal injection of MSU crystals did not induce systemic inflammatory response in rats

**Figure 5:**
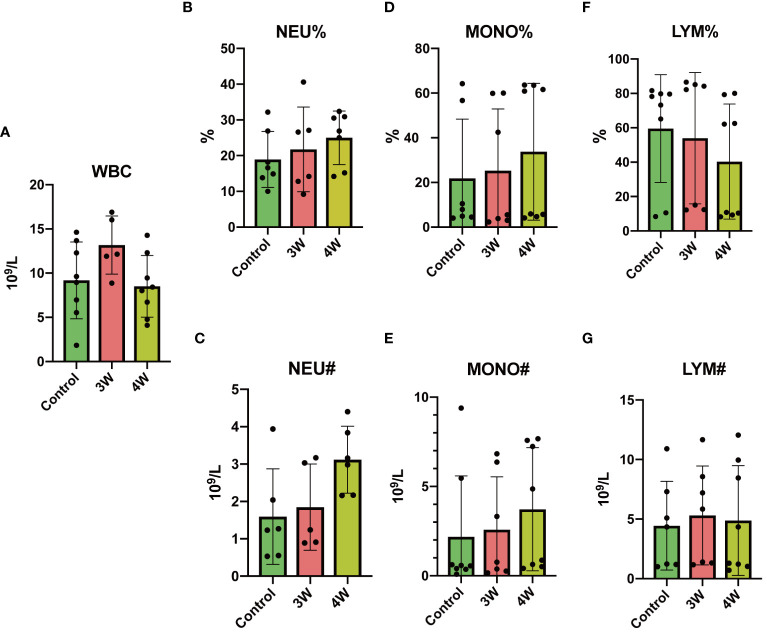
Intrarenal administration of MSU crystals did not induce a systemic inflammatory response in rats. The percentage and total number of inflammatory cells per liter in the blood of rat was analyzed using a fully automated blood cell analyzer. **(A)** Blood leukocyte (WBC) count (109/L). **(B)** Blood neutrophil (NEU) percentage (%). **(C)** Blood neutrophil count(109/L). **(D)** Blood monocyte (MONO) percentage (%). **(E)** Blood monocyte count (109/L). **(F)** Blood lymphocyte (LYM) percentage (%). **(G)** Blood lymphocyte count (109/L). Blood samples from rata were collected three or four weeks (3W/4W) after injection of suspension of MSU crystals in the upper pole and after injection of PBS in the lower pole of the rat kidney.

To know the impact of intrarenal injection with MSU crystals on systemic inflammatory response we conducted a thorough analysis of inflammatory cell types in rat blood samples. The results of flow cytometry analyses revealed almost no significant differences in counts or proportions of leukocyte, neutrophil, lymphocyte, and monocyte in the circulation during the third and fourth weeks post-injection compared to the control group (**Figure 5**). The results suggest that the intrarenal injection of MSU crystals did not cause any infection or induce systemic inflammatory response in the rats.

### Intrarenal Injection of MSU crystals induces focal renal interstitial fibrosis

**Figure 6:**
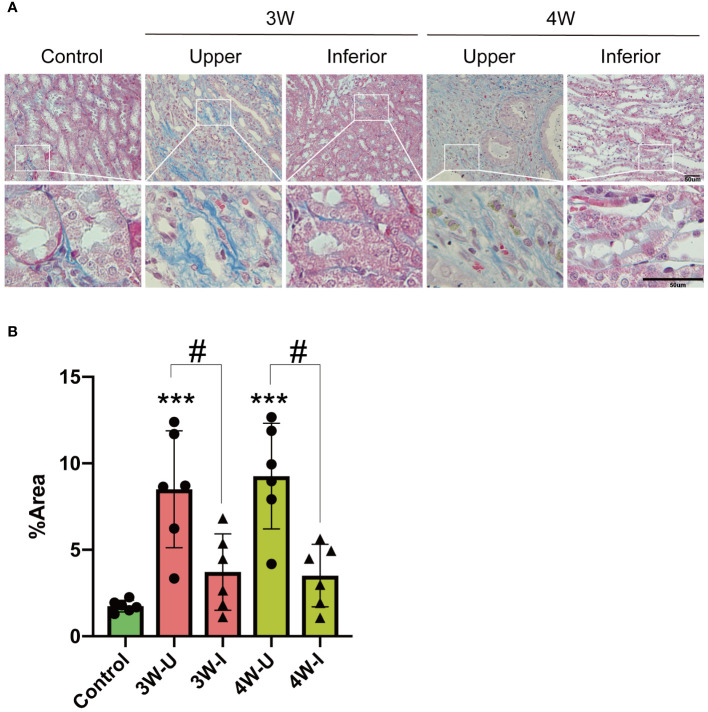
Intrarenal injection of MSU crystals induces focal renal interstitial fibrosis as revealed by Masson (Trichrome) staining of rat kidney sections. **(A)** Masson (Trichrome)staining of connective tissue fibers and collagen in the kidney sections as shown by positively stained (blue) area of rat kidney sections (n = 6; mean ± SEM). Scale bar = 50 μm. **(B)** Quantification of the area of Masson (Trichrome) stained blue region (n = 5–6; mean ± SEM). Rat kidneys were sectioned three or four weeks (3W/4W) after injection of suspension of MSU crystals in the upper pole (3W-U/4W-U) and PBS injection in the lower pole (3W-I/4W-I) of the rat kidney. # P < 0.05, *** P < 0.001.

The MSU crystal-induced nephropathy was previously shown as the result of stimulation of renal inflammation and fibrosis through activation of the cytosolic Nod-like receptor protein 3 (NLRP3) inflammasome (12). We assessed the effect of intrarenal MSU crystal injection on renal fibrosis by Masson’s Trichrome staining of connective tissue fibers and collagen in renal tissue sections and measured the positively stained (blue) area. The results show a significant increase in fibrosis in the renal interstitium at the upper pole of kidneys injected with MSU crystals during the third and fourth weeks, compared to the lower pole injected with PBS and control rat kidneys. We did not find any significant increase in fibrosis at the PBS-injected lower pole of the kidneys at weeks 3 and 4 compared to the control rats (**Figure 6**). The results are indicative of the effect of MSU crystals on localized induction of interstitial fibrosis only at the site of MSU crystal injection in kidney during the third and fourth weeks.

### Intrarenal injection of MSU crystals results in renal impairment in rats without influencing blood uric acid levels

**Figure 7:**
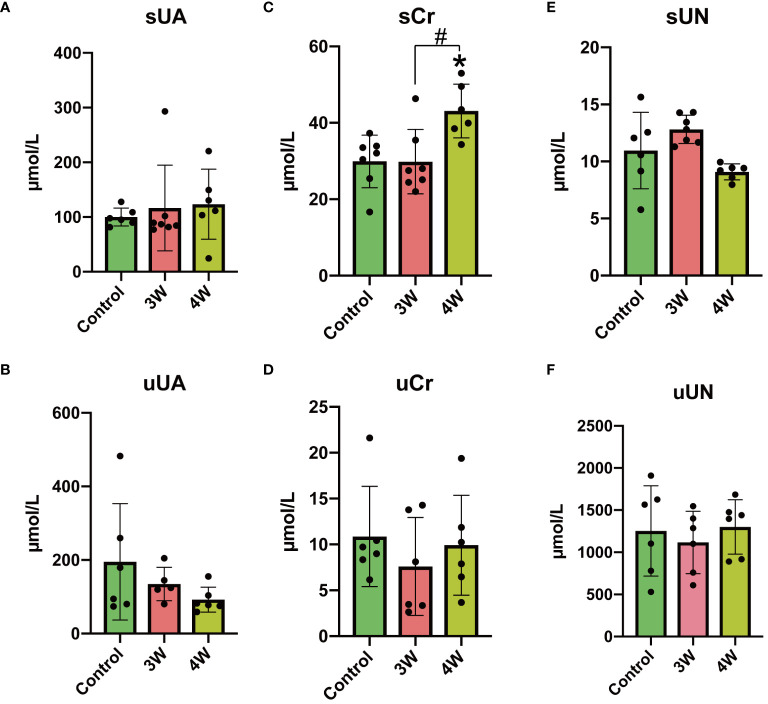
Effect of intrarenal injection of MSU crystals on serum and urinary indices of renal function in rats. **(A)** Serum urate (sUA) (ummol/L). **(B)** Urinary uric acid (uUA) (ummol/L). **(C)** Serum creatinine (sCr) (ummol/L). **(D)** Urinary creatinine (uCr)(ummol/L). **(E)** Serum urea nitrogen (sUN) (ummol/L). **(F)** Urinary urea nitrogen (uUN) (ummol/L).*, # P < 0.05.

To assess the effect of intrarenal injection of MSU crystals on rat kidney function, we measured the levels of uric acid, creatinine, and urea nitrogen in urine and serum. Comparative analysis revealed no significant differences in uric acid and urea nitrogen levels in rat serum and urine three and four weeks after MSU injection compared to control rats (**Figures 7A, B, E, F**). However, the serum creatinine levels showed a significant increase four weeks after the MSU injection compared to the control group, while no noticeable changes were observed in urine creatinine (**Figures 7C, D**). These results indicate that the intrarenal injection of MSU crystals did not interfere with the renal function related to uric acid transport (reabsorption/secretion) or metabolism. However, the injection of MSU crystals significantly increased serum creatinine (a waste product of the muscles) indicating MSU crystals might have triggered renal dysfunction in rats by the fourth week post-injection.

### Both MSU crystals and soluble UA results in renal impairment, pathological damage and fibrosis

**Figure 8:**
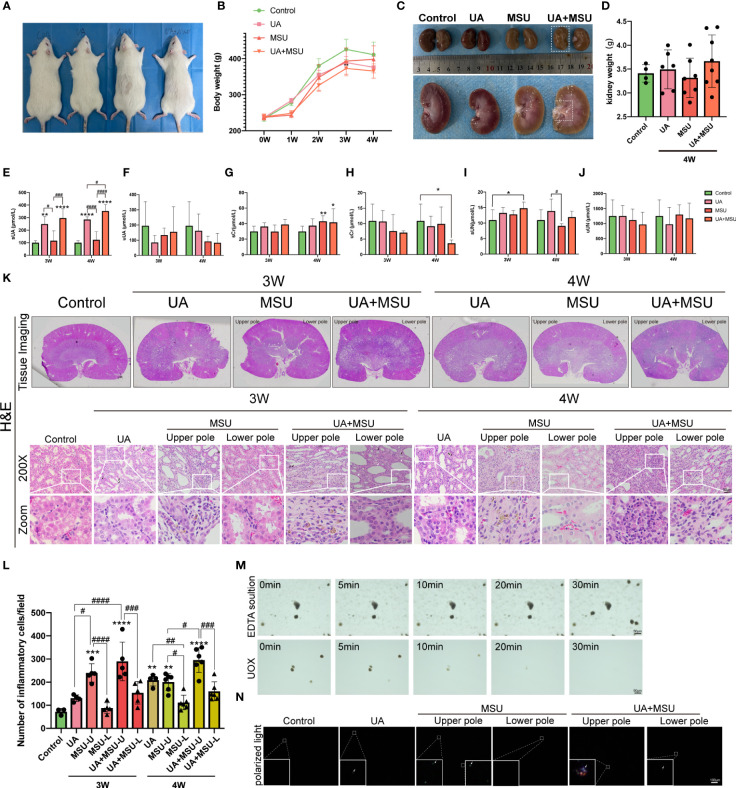
Intrarenal injection of MSU crystals into the kidneys of normal rats or PO-induced hyperuricemic rats triggers renal impairment, pathological damage and inflammatory cell infiltration. **(A)** The pictures of rats from left to right are the representative control rat, PO-induced hyperuricemic rat (of UA group), MSU crystals-injected rat (of MSU group), and MSU crystals-injected PO-induced hyperuricemic rat (of UA+MSU group). These pictures were taken four weeks (4W) after injection of suspension of MSU crystals (MSU group). The PO-induced group of hyperuricemic rats (UA group) was generated by intragastric gavage of 750 mg/kg of PO and 300 mg/kg of UA. The UA+MSU group of rats was generated by intragastric gavage of 750 mg/kg of PO and 300 mg/kg of UA after intrarenal injection of MSU crystals. **(B)** Whole body weight (g) of rats of the control, UA, MSU, and UA+MSU groups were measured from zero to four weeks after MSU injection/gavage. **(C)** The pictures show the physical appearance of rat kidneys in the control, UA, MSU and UA+MSU groups taken at four weeks after after MSU injection/gavage. **(D)** The weight (in grams) of the kidneys of the rats was measured four weeks after MSU injection/gavage. **(E)** Serum urate (sUA) (ummol/L); **(F)** Urinary uric acid (uUA) (ummol/L); **(G)** Serum creatinine (sCr) (ummol/L); **(H)** Urinary creatinine (uCr)(ummol/L); **(I)** Serum urea nitrogen (sUN) (ummol/L) and **(J)** Urinary urea nitrogen (uUN) (ummol/L). **(K)** Hematoxylin and Eosin (H&E) staining of a representative of the kidney sections (n = 5–8; mean ± SEM) of control, UA, MSU and UA+MSU groups. Tissue Imaging and 200x field of view. Scale bar = 2μm, 50 μm. Arrow sign (→) on the picture shows damage to the peripheral renal tubules; the white arrow indicates tubular dilatation, while the black arrow denotes vacuolar degeneration. **(L)** Inflammatory cell count infiltrated into the interstitium of rat kidneys three or four weeks (3W/4W) after MSU injection/gavage. (n = 5–6; mean ± SEM). The panoramic scanning of slides of kidney sections stained for multiplex fluorescence immunohistochemistry with TSA-488 dye conjugated NLRP3 antibody, TSA-555 dye conjugated F4/80 antibody (for labeling matured macrophages), and TSA-647 dye conjugated CD11b antibody (for labeling monocytes, activated lymphocytes, macrophages) was used for counting the number of infiltrated inflammatory cells. **(M)** The intrarenally accumulated MSU crystals were shown getting dissolved by UOX (urate oxidas’uricase) treatment for about 10–30 min. The EDTA solution used to make UOX solution had no effect in dissolving MSU crystals at the site of injection. The picture was taken at 200X magnification. Scale bar, 50μm. **(N)** The photograph of rat kidney sections was taken using a polarized light microscope. Scale bar,100μm. The white arrow symbol (→) shows the presence of MSU Crystals. *, # <0.05; **, ##P < 0.01; ***, ###P < 0.001; ****, ####P < 0.0001.

To know the difference in the impacts of MSU crystals (MSU group) and soluble UA on renal injury, we induced hyperuricemia in rats by gavaging potassium oxonate (PO) and UA (UA group) to simulate the effect of soluble UA on the kidney. We also used rats with hyperuricemia (by gavaging PO and UA) after intrarenal injection of MSU crystals (UA+MSU group) to simulate the effects on the kidney when MSU crystals and soluble uric acid are present in the kidney at the same time (**Figures 1A, B**). The outward appearance of the rat kidneys and the mass were compared across four different groups. The results demonstrate that the UA, MSU, and UA+MSU groups exhibited no significant differences in physical appearance (**Figure 8A**), whole body weight (**Figure 8B**), or overall appearance (**Figure 8C**) or weight (**Figure 8D**) of the kidneys compared to controls during the third and fourth weeks. Thus, in the presence of MSU crystals, soluble UA and both in the kidney, the overall health conditions of rats did not change after the third and fourth weeks post-procedure. A comparative analysis of kidney function indicators revealed that at the third and fourth weeks of modelling, there was no significant difference in serum UA (sUA) levels between the MSU and control groups. Nevertheless, sUA levels were found to be significantly elevated in the UA and UA+MSU groups in comparison to the control and MSU groups. Furthermore, at the fourth week, sUA levels were found to be significantly higher in the UA+MSU group than in the UA group (**Figure 8E**). There was no significant difference in serum creatinine (sCr) levels between the UA group and the control group at week four. However, sCr levels were significantly higher in the MSU group and the UA+MSU group (**Figure 8G**). The modelling groups exhibited no significant differences in urine UA (uUA), urine creatinine (uCr), serum urea nitrogen (sUN) and urine urea nitrogen (uUN) compared to the control group at weeks three and four (**Figures 8F, H–J**). The results indicate that the administration of PO and UA by gavage may affect renal function related to uric acid transport (reabsorption/secretion) or metabolism. Furthermore, the presence of MSU crystals in the kidney appears to exacerbate this effect. Concurrently, the injection of MSU crystals into the kidneys of PO and UA gavaged rats resulted in a significant elevation in serum creatinine (a byproduct of muscle), indicating that MSU crystals may be the primary contributor to renal dysfunction in rats during the fourth week following injection, rather than soluble UA.

The results of hematoxylin and eosin (H&E) staining show that the UA group, UA+MSU group, and the upper pole of the kidney in MSU groups suffered significant damage, as evidenced by tubular dilatation and vacuolar degeneration (shown by the arrows in the **Figure 8K**). In contrast, the lower pole of the kidney in the MSU group did not exhibit any significant pathological changes or damages compared to the control group after third and fourth weeks of the procedures. It is noteworthy that pathological damage in the MSU group was present only in the vicinity of the MSU crystals. In contrast, pathological damage in the UA and UA+MSU groups was observed diffused throughout the renal tissue (**Figure 8K**). To know the mechanism how MSU crystals and soluble UA induce pathological changes in the renal tissue, the number of inflammatory cells infiltrating the interstitium of renal tissue was quantified (**Figure 8L**). The results show a significant increase in the infiltration of inflammatory cells in the third and fourth weeks after procedures in the kidneys of UA group, UA+MSU group, and the upper pole of the kidney in the MSU groups compared with control group. Furthermore, the number of inflammatory cells was significantly increased in the upper pole of the kidney in the MSU group and the UA+MSU group in the presence of MSU crystals compared to the lower pole of the kidney injected with PBS.

To demonstrate that intrarenal injection of MSU crystals results in kidney damage, we employed uric acid oxidase (UOX)to dissolve the intrarenally injected MSU and used polarized light microscopy to observe the kidney tissue after injection. The results indicated that uricase could dissolve the intrarenally injected MSU and within 30 minutes, while the dissolution reagent without UOX was ineffective (**Figure 8M**). Interestingly, the results of polarized light microscopy of rat kidney sections also showed the increased growth/size/number of the injected MSU crystals in the kidney of UA+MSU group of rats (**Figure 8N**). This indicates that under condition of hyperuricemia, intrarenally injected smaller MSU crystals acted like seeding crystals to accelerate the growth of the injected smaller MSU crystals that ultimately results in more damages to the kidney.

**Figure 9:**
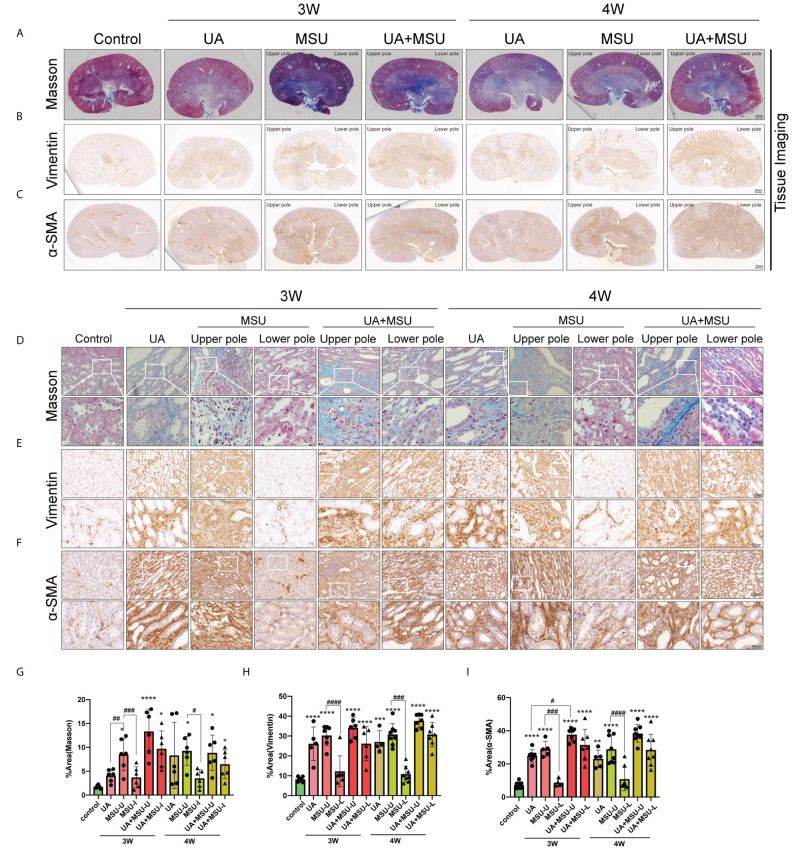
Intrarenal injection of MSU crystals into the kidneys of normal rats or PO-induced hyperuricemic rats induces renal interstitial fibrosis, as evidenced by Masson’s trichrome staining (for detection of the extent of collagen fibers deposition indicative of fibrosis) and immune-staining of α-SMA (α-smooth muscle actin, a marker of fibrosis) and vimentin (a cytoskeletal protein, a marker of fibrosis) in rat kidney sections. **(A)** Images of kidney sections taken after Masson’s trichrome staining. Scale bar = 2μm. Muscle fibers are stained red and collagens are stained blue. **(B)** Images of kidney sections taken after immune staining using anti-Vimentin antibody, HRP-linked secondary antibody and DAB+ [3,3’-diaminobenzidine (DAB+) substratechromogen which results in a brown-colored precipitate at the antigen site. Scale bar = 2μm. **(C)** Images of kidney sections taken after immune staining using anti-α-SMA antibody, HRP-linked secondary antibody and the above-mentioned substrate-chromogen. Scale bar = 2μm. **(D)** Representative images of kidney sections taken after Masson’s trichrome staining of connective tissue fibers particularly collagen (blue) (n = 5–8; mean ± SEM). Scale bar = 50 μm. **(E)** Representative images of kidney sections taken after immune-staining using anti-vimentin antibody (brown) (n = 5–8; mean ± SEM). Scale bar = 50 μm. **(F)** Representative images of kidney sections taken after immune-staining using anti-α-SMA antibody (brown) (n = 5–8; mean ± SEM). Scale bar = 50 μm. **(G)** Quantification of the Masson’s trichrome stained blue region (for collagen fibers deposition) (n = 5–8; mean ± SEM). **(H)** Quantification of the of brown-stained area for Vimentin expression (n = 5–8; mean ± SEM). **(I)** Quantification of the brown-stained area for α-SMA expression (n = 5–8; mean ± SEM). Rat kidneys were sectioned three or four weeks (3W/4W) after MSU injection/gavage. *, # <0.05; **, ## P < 0.01; ***, ### P < 0.001; ****, #### P < 0.0001.

Next, we stepped forward to know the differences in the impacts of intrarenal MSU crystals, soluble UA, and UA+MSU on the degree of renal fibrosis through evaluation of the fibrosis marker proteins α-SMA and vimentin proteins of connective tissue fibers in renal tissue sections using Masson’s trichrome staining and immunohistochemical staining. The results show significant degree of fibrosis, in the renal tissues of the UA and UA+MSU groups, as well as in the upper pole of the kidneys in the MSU group at the location of MSU crystal injection (**Figures 9A–F**). Quantitative analyses reveal that the UA group, the UA+MSU group, and the upper pole of the kidney in the MSU group exhibits significantly higher level of Vimentin and α-SMA than the control group (**Figures 9G–I**). In contrast, no significant difference was observed in the positive area of the three indicators in the lower pole of the kidney in the MSU group compared to the control group (**Figures 9G–I**). The results thus indicate that MSU crystals, soluble UA, and their combination can induce interstitial fibrosis during the third and fourth weeks.

### MSU crystals induce NLRP3 activation in renal tissue monocytes and macrophages and elevate serum IL-1β levels

MSU crystals have been demonstrated to release inflammatory factors by activating the Nod-like receptor protein 3 (NLRP3) in monocytes and macrophages, thereby exacerbating the tissue inflammatory response (12). In order to ascertain the role of MSU crystals and soluble UA in the regulation of inflammatory factors in renal tissues, kidney tissue sections were stained using multiplex immunohistochemical staining. Green signals represented NLRP3, red signals represented F4/80 (a marker for macrophages) and pink signals represented CD11b (a marker for monocytes). The results demonstrated that there was no significant difference in the CD11b-positive area between the UA, MSU and UA+MSU groups (**Figures 4A, B**). The F4/80-positive area was significantly greater in the UA group, MSU group and UA+MSU group compared to the control group. Furthermore, the F4/80-positive area of the upper pole of the kidney injected with MSU crystals was significantly greater than that of the lower pole injected with PBS in the MSU group and UA+MSU group (**Figures 4A, C**). The number of cells that were positive for both NLRP3 and CD11b or F4/80 was counted. The results reveal a remarkable increase in the number of NLRP3, CD11b and F4/80-positive cells in the MSU and UA+MSU groups in comparison to the control group. A significant increase in the number of CD11b and F4/80-positive cells was also observed in the PBS-injected lower pole of the kidney in the UA+MSU group compared to the PBS-injected lower pole of the kidney of MSU group of rats (**Figures 4A–E**). Since, NLRP3 activation in macrophages and monocytes was reported to increase IL-1β secretion (12), we examined the level of IL-1β in the blood of all these experimental groups of rats. The results show that the level of IL-1β in the blood of UA group, MSU group and the UA+MSU group was significantly increased compared to that of the control group. (**Figure 4F**) The results thus indicate that the intrarenal injection of MSU crystals may be the causal factor to the observed increase in IL-1β expression.

## Discussion

GN is caused by the precipitation of MSU crystals in the kidney tubules, usually collecting ducts with tubular injury (1). Patients suffering from acute GN usually have severe hyperuricemia, gout and kidney failure and/or impaired kidney function with renal fibrosis (1, 2). In this study, we found that direct administration of the suspension of MSU crystals into the rat kidney caused significant infiltration of inflammatory cells and interstitial fibrosis at the site of injection, while the other uninjected sites or sites injected with PBS remained almost normal. However, we can’t rule out the impact of MSU crystals on other sites of kidney over longer periods of time.

Previous studies on GN have mainly relied on models that had elevated levels of serum urate (11, 13). In contrast, this study design aimed to explore the localized effect of MSU crystals in the rat kidney compared with the other parts of the kidney. We monitored the presence of MSU crystals at the site of injection directly by light microscopy to confirm direct effect of MSU crystals on infiltration of inflammatory cells and fibrosis. However, in other previous studies, confirming the presence of MSU crystals at the site of infiltration of inflammatory cells and fibrosis was a challenging issue to prove that the symptoms of GN was caused directly by MSU crystals, not by circulating higher level of urate (11).

To assess the role of only the MSU crystals on the pathogenesis of GN, it is important to exclude the interference caused by elevated levels of circulating uric acid, which can be very challenging. In this study, renal inflammation, fibrosis and tubular injury was induced by the MSU crystals (detectable at the site of injection directly by light microscopy) while keeping the serum urate at normal level during the study period. Furthermore, by comparing this study model of GN with the GN model having elevated uric acid levels, it might be possible to simulate renal injury in patients with clinically comorbid hyperuricemia and gout. The use of this study approach can be helpful to understand the differences between the pathogenic mechanisms of HN and GN.

GN is characterized by the presence of mostly MSU crystals in the renal tubules usually collecting ducts with tubular injury1 and/or interstitium, with infiltrating inflammatory cells such as macrophages, lymphocytes, and granulocytes in the interstitium of kidneys (2, 14) accompanied by fibrosis. The results of this study confirm that the presence of MSU crystals at the site of injection in the rat kidney caused profound infiltration of inflammatory cells accompanied by fibrosis without affecting other areas of kidney injected with PBS. In this study, serum urate levels remained almost unaffected in both serum and urine in the MSU crystals injected rats like the control group. Thus, the induction of GN symptoms and renal injury by MSU crystals in this study, unlike that in the HUA model, was under the conditions of normal serum urate levels. Regrettably, our study was not designed for a longer observation period to determine the pathological development of rat kidney tissue after MSU crystal injection at 4 weeks. Therefore, extending the observation time after injection might bring some data valuable for the mechanism of pathological progression of disease.

A comparison of renal function indexes, pathological damage and fibrosis in rats subjected to different modelling methods revealed that both MSU crystals and soluble UA in the kidneys are associated with renal impairment, pathological damage and fibrosis. Furthermore, these factors appear to act in a synergistic manner, contributing to the pathogenesis of renal disease. However, the indexes of renal impairment were not identical. MSU crystals primarily affected the excretion or production of sCr, whereas PO and UA gavage primarily affected the excretion or production of sUA, resulting in elevated sUA levels. This is attributed to the fact that PO is a selective competitive inhibitor of uricase/urate oxidase (EC 1.7. 3.3) that causes hyperuricemia by blocking the conversion of uric acid to more soluble allantoin (10). Furthermore, the renal pathology and fibrosis induced by MSU injection only at a specific site of kidney differ from that induced by PO. MSU crystals induce tissue damage, inflammatory cell infiltration, and fibrosis at the site of their action, whereas in PO-induced hyperuricemia, soluble UA induces tissue damage, inflammatory cell infiltration, and fibrosis in all over the kidney KO can cause hyperkalemia which may cause unexpected animal death due to heart failure (15, 16). It is noteworthy that the combined intrarenal injection of MSU and gavage feeding of PO approach resulted in further exacerbation of some renal function parameters, renal pathology and fibrosis. Our results are consistent with recent findings suggesting that soluble UA can trigger renal injury as well as MSU crystals (7, 8). In our study, we administered 750 mg/kg PO and 300 mg/kg UA by gavage, resulting in sUA levels of approximately 250–280 umol/L at weeks 3 and 4. This indicates that this concentration of soluble UA may act as a pro-inflammatory or pathologically damaging agent.

It is believed that the activation of immune cells, primarily macrophages and monocytes, is the primary cause of the inflammatory response induced by MSU crystals (5). The results of our investigation indicate that the intrarenal injection of MSU crystals can result in the infiltration of macrophages into renal tissues and the activation of NLRP3 in macrophages and monocytes. In this study, we did not find any significant effect of injected intrarenal MSU crystals on infiltration of monocytes, which could be due to the imperfect choice of CD11b as the proper marker of the monocytes in renal tissues at this stage of the disease. It has been demonstrated in the literature that monocytes exhibit distinct subtypes of expression at different stages of gout (17), suggesting that future study with appropriate selection markers for staining of different populations of monocytes might give us better conclusive results. Activation of NLRP3 in macrophages and monocytes releases IL-1β (12), which amplifies the inflammatory response. The results indicate that blood levels of IL-1β may be elevated following intrarenal MSU crystal deposition. However, further validation is necessary to ascertain whether the increased IL-1β originates from activated macrophages and monocytes. Furthermore, MSU crystals have been observed to influence the activity of a range of immune cells, thus, the present study might act as the basis for further detailed study identifying and associating which population of infiltrating inflammatory cells in the renal interstitium, are the major players of crystal-induced renal injury and what kind of proinflammatory cytokines they secrete into the extracellular space.

In conclusion, we were able to induce the symptoms of gouty nephropathy in a rat model by intrarenal injection of the suspension of MSU crystals. We verified that the stable presence of MSU crystals (in absence of hyperuricemia) in renal tissue caused infiltration of inflammatory cells accompanied by interstitial fibrosis and tubular injury localized at the site of injection without affecting the other areas of the kidney injected with PBS. Renal impairment was observed four weeks after MSU crystal injection. This strategic study design is effective for studying gouty nephropathy triggered by MSU crystals only, as it isolates from the impact of elevated level of circulating urate.

## Data availability statement

The original contributions presented in the study are included in the article/supplementary material. Further inquiries can be directed to the corresponding authors.

## Ethics statement

The animal study was approved by Welfare Committee of Guangdong Academy of Chinese Medicine (Approval Document No. 2022051). The study was conducted in accordance with the local legislation and institutional requirements.

## Author contributions

DLL: Conceptualization, Data curation, Visualization, Writing – original draft, Writing – review & editing. YL: Validation, Visualization, Writing – review & editing. XC: Visualization, Writing – review & editing. JO: Visualization, Writing – review & editing. DYL: Validation, Writing – review & editing. QW: Validation, Writing – review & editing. XF: Writing – review & editing. HQ: Validation, Writing – review & editing. XW: Writing – review & editing. SW: Writing – review & editing. SY: Writing – review & editing. AL: Writing – review & editing. JZ: Writing – review & editing. XL: Writing – review & editing. GZ: Writing – review & editing. CL: Funding acquisition, Writing – review & editing. WM: Funding acquisition, Writing – review & editing.
